# P37 Variable adherence to and effectiveness of a vancomycin continuous infusion protocol within ICUs at a London tertiary-care hospital: a single-centre retrospective service evaluation

**DOI:** 10.1093/jacamr/dlac004.036

**Published:** 2022-02-16

**Authors:** Robert Oakley, Prijay Bakrania, Ting Yau, Joseph Standing, Dagan Lonsdale

**Affiliations:** St George’s University Hospitals NHS Foundation Trust, London, UK; Guy’s and St Thomas’ NHS Foundation Trust, London, UK; St George’s University Hospitals NHS Foundation Trust, London, UK; University College London, London, UK; St George’s University Hospitals NHS Foundation Trust, London, UK; St George’s, University of London, London, UK

## Abstract

**Background:**

Appropriate vancomycin dosing and therapeutic monitoring is important to optimize treatment for serious Gram-positive infections. In St George's Hospital ICUs, the treatment protocol is a loading dose (<65 kg, 1000 mg; ≥65 kg, 1500 mg), followed by a continuous infusion based on creatinine clearance (CL_CR_). Vancomycin serum concentrations are taken daily at 0600 hours (target therapeutic range 20–25 mg/L). Non-therapeutic concentrations may negatively affect clinical outcomes and prolong length of stay.^1^

**Methods:**

Electronic prescribing and medicine administration data (vancomycin dose, dose timings, indication, patient biochemistry and demographics) within a 66-bed ICU service were reviewed retrospectively (July 2020–July 2021). Cockcroft-Gault CL_CR_ was calculated using total body weight (TBW), or adjusted body weight in obese^1^ patients.

**Standards:**

(i) Proportion of patients with a therapeutic concentration within two serum samples; and (ii) time taken to achieve therapeutic concentration.

**Results:**

Ninety-four percent (51/54) of patients received a correct protocol instructed loading dose, 31% (17/54) received a correct loading and maintenance dose, and 70% (38/54) had a vancomycin serum concentration taken. Forty-one percent (7/17) of correctly protocol dosed and monitored patients were therapeutic at their first day serum concentration, which decreased to 20% (3/15) by their second day serum concentration. Consistently therapeutically dosed patient groups included obese and normal weight patients with CL_CR_ 10–20  mL/min and 21–50  mL/min. Whereas underweight and obese patients with CL_CR_ >50  mL/min and 21–50  mL/min became supratherapeutic by their second day serum concentration. Obese and normal weight patients with CL_CR_ >50  mL/min were consistently subtherapeutic. The mean time taken for non-therapeutic patients who continued treatment (*n = *7) to become therapeutic after dose adjustments was 4 days. Patients (*n = *17) were 82% male and of a mean age of 62 ± 17 years. Mean TBW was 79 ± 23 kg and mean CL_CR_ 65 ± 48  mL/min. Sepsis was the most common vancomycin indication (65%).

**Conclusions:**

Multiple drug recording formats, plus adherence to and dosing of different aspects of the vancomycin protocol, requires review. This is to ensure accurate vancomycin administration documentation and that therapeutic concentrations are achieved more rapidly and consistently, whilst minimizing toxicity. A quality improvement project has been instigated to focus on protocol education, accessibility and improving IT infrastructure. Increased patient volume of distribution and renal function variability within the ICU population adds complexity to vancomycin pharmacokinetics. Research into pharmacokinetic models better representing the local ICU population and AUC_24_ drug monitoring is being explored based on recent international guidance.^1^
